# Pilot study comparing a new virtual reality–based visual field test to standard perimetry in children

**DOI:** 10.1016/j.jaapos.2024.103933

**Published:** 2024-05-08

**Authors:** Yeabsira Mesfin, Alan Kong, Benjamin T. Backus, Michael Deiner, Yvonne Ou, Julius T. Oatts

**Affiliations:** a Department of Ophthalmology, UCSF School of Medicine, San Francisco, California; b Vivid Vision Inc, San Francisco, California

## Abstract

**PURPOSE:**

To assess the feasibility and performance of Vivid Vision Perimetry (VVP), a new virtual reality (VR)–based visual field platform.

**METHODS:**

Children 7–18 years of age with visual acuity of 20/80 or better undergoing Humphrey visual field (HVF) testing were recruited to perform VVP, a VR-based test that uses suprathreshold stimuli to test 54 field locations and calculates a fraction seen score. Pearson correlation coefficients were calculated to evaluate correlation between HVF mean sensitivity and VVP mean fraction seen scores. Participants were surveyed regarding their experience.

**RESULTS:**

A total of 37 eyes of 23 participants (average age, 12.9 ± 3.1 years; 48% female) were included. All participants successfully completed VVP testing. Diagnoses included glaucoma (12), glaucoma suspect (7), steroid-induced ocular hypertension (3), and craniopharyngioma (1). Sixteen participants had prior HVF experience, and none had prior VVP experience, although 7 had previously used VR. Of the 23 HVF tests performed, 9 (39%) were unreliable due to fixation losses, false positives, or false negatives. Similarly, 35% of VVP tests were unreliable (as defined by accuracy of blind spot detection). Excluding unreliable HVF tests, the correlation between HVF average mean sensitivity and VVP mean fraction seen score was 0.48 (*P* = 0.02; 95% CI, 0.09–0.74). When asked about preference for the VVP or HVF examination, all participants favored the VVP, and 70% were “very satisfied” with VVP.

**CONCLUSIONS:**

In our cohort of 23 pediatric subjects, VVP proved to be a clinically feasible VR-based visual field testing, which was uniformly preferred over HVF.

Many ophthalmic diseases in children cause visual field (VF) defects. Identifying the location, pattern, severity, and progression of VF defects assists in the diagnosis and treatment of the underlying pathology.^1^ The current gold standard for formal VF testing is standard automated perimetry (SAP), commonly performed using Humphrey visual field (HVF) testing. This type of perimetry requires a high level of cooperation: the patient’s head must be placed against a stationary headrest, with one eye occluded and the other eye maintaining central fixation for the entirety of the 5- to 10-minute test. Because many children have trouble maintaining appropriate fixation or positioning during the test,^2^ there has been increased interest in developing child-friendly ways to perform VF testing.

Vivid Vision Perimetry (VVP; Vivid Vision, San Francisco, CA) is a novel software that utilizes off-the-shelf virtual reality (VR) headsets to perform perimetry. With the headset in place, stimuli are presented to each eye sequentially. First, the subject is asked to move their head to move the cursor to the location of a fixation target. Once this is located, a stimulus is presented in a different location. If the examinee moves their head to move the cursor to that location, the stimulus is registered as “seen.” This fixation task and stimulus presentation is repeated for 54 points across 24° of the VF. Unlike HVF testing, VVP uses a multifixation strategy to encourage foveation.^3^ Prior VVP studies have demonstrated high test-retest reliability and correlations with standard automated perimetry in adults (Greenfield JA, et al. IOVS 2020;61:ARVO eAbstract 4800).^4^ This pilot study aimed to evaluate the feasibility and performance of VVP in a pediatric population.

## Subjects and Methods

This study was approved by the University of California, San Francisco (UCSF) Institutional Review Board, adhered to the tenets of the Declaration of Helsinki, and complied with all requirements of the US Health Insurance Portability and Accountability Act of 1996. Pediatric patients scheduled for routine HVF testing as part of their standard clinical care were recruited from the UCSF Ophthalmology Clinic. Written parental consent and verbal child assent were obtained. Inclusion criteria were age 7–18 years, ability to follow directions, and best-corrected visual acuity of 20/80 or better. Patients with spherical refractive errors exceeding ± 6.0 D or those with impairments that hindered their ability to complete testing with VVP or HVF were excluded. Patients with only one qualifying eye were included, although the VVP test was administered only to the qualifying eye. Given the reliance of the VR test on binocularity, patients with constant, manifest strabismus were included, but only their fixing eye was tested.

### Visual Field Testing

**FIG 1.**
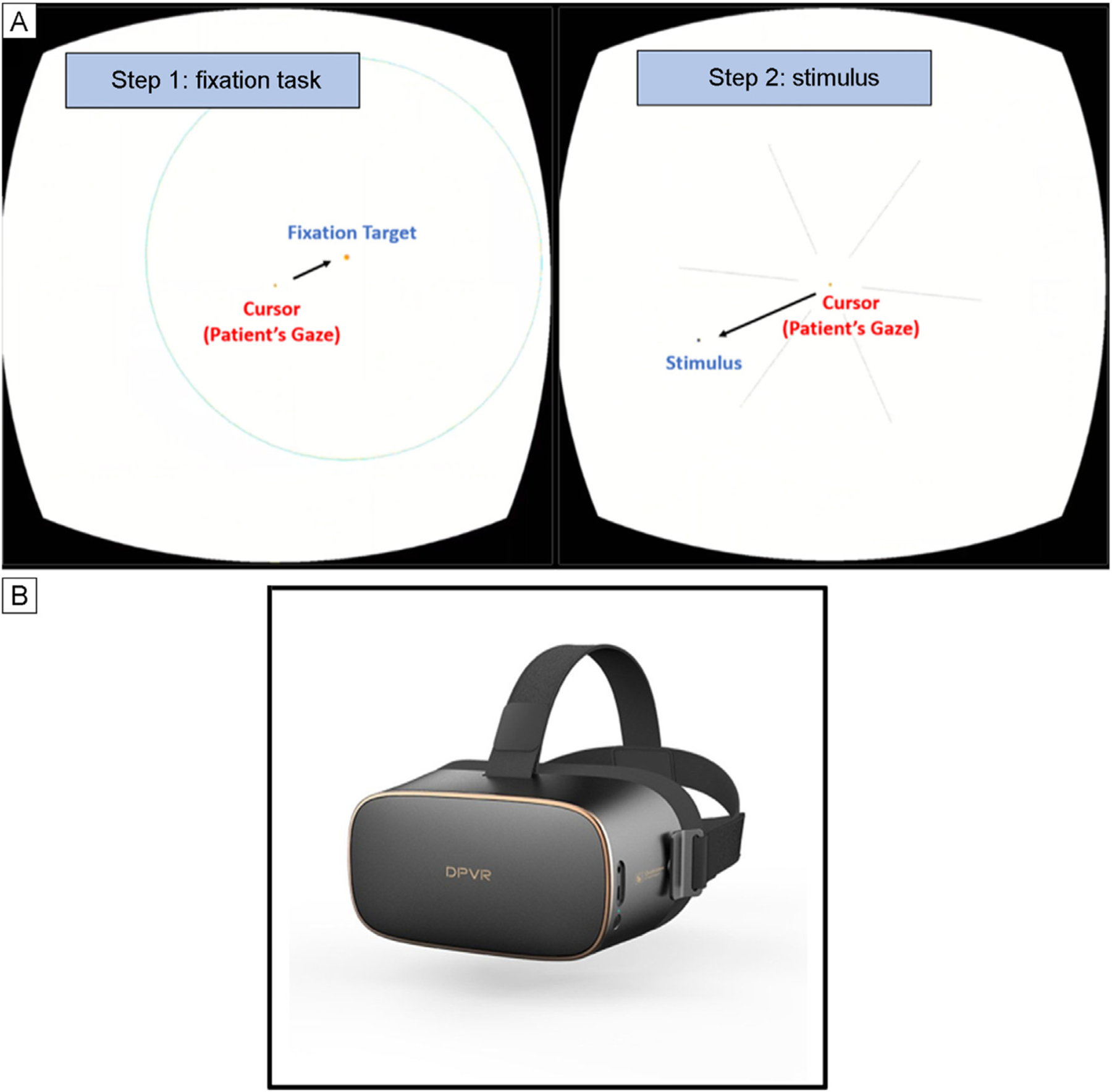
A, Schematic demonstrating the fixation and stimulus task presented during Vivid Vision Perimetry (VVP) examination. Patients are instructed to complete a fixation task in the first step. After, a stimulus is presented in one of six segments, and the patient is instructed to use head motion to move the stimulus into the segment where they perceived the stimulus. B, DPVR P1 Pro headsets used to administer the VVP examination.

All patients performed HVF testing first followed by VVP. HVF testing was performed using a standard Humphrey Field Analyzer device with either the 24-2 SITA Fast or Standard protocol (Swedish Interactive Testing Algorithm; Zeiss, Dublin, CA). Prior to HVF testing, patients were provided instructions by a trained ophthalmic technician. HVF tests with false positive rate <30%, false negative rate <30%, and fixation losses <20% were considered reliable. VVP testing was performed using a VR headset (DPVR P1 Pro, Shanghai, China; Figure 1) with the VVP 24-2 Swift protocol (VVP Swift) installed. VVP Swift tests both eyes in a single session using randomly alternating stimuli to each eye. If prespecified, the software can also perform monocular testing. In contrast to HVF which is a threshold test using a white stimulus on a white background, VVP Swift is a suprathreshold test that uses black stimuli on a white background. Previous studies have demonstrated the utility of using dark stimuli to detect visual field loss with other testing platforms.^5^ This suprathreshold test presents small black round spots (diameter = 0.43°) for 300 ms against a white background of luminance 25 cd/m^2^. Before VVP testing, a team member instructed the participant on using the VVP headset, then started a prerecorded tutorial installed on the headset. The test began immediately after the tutorial.

**FIG 2.**
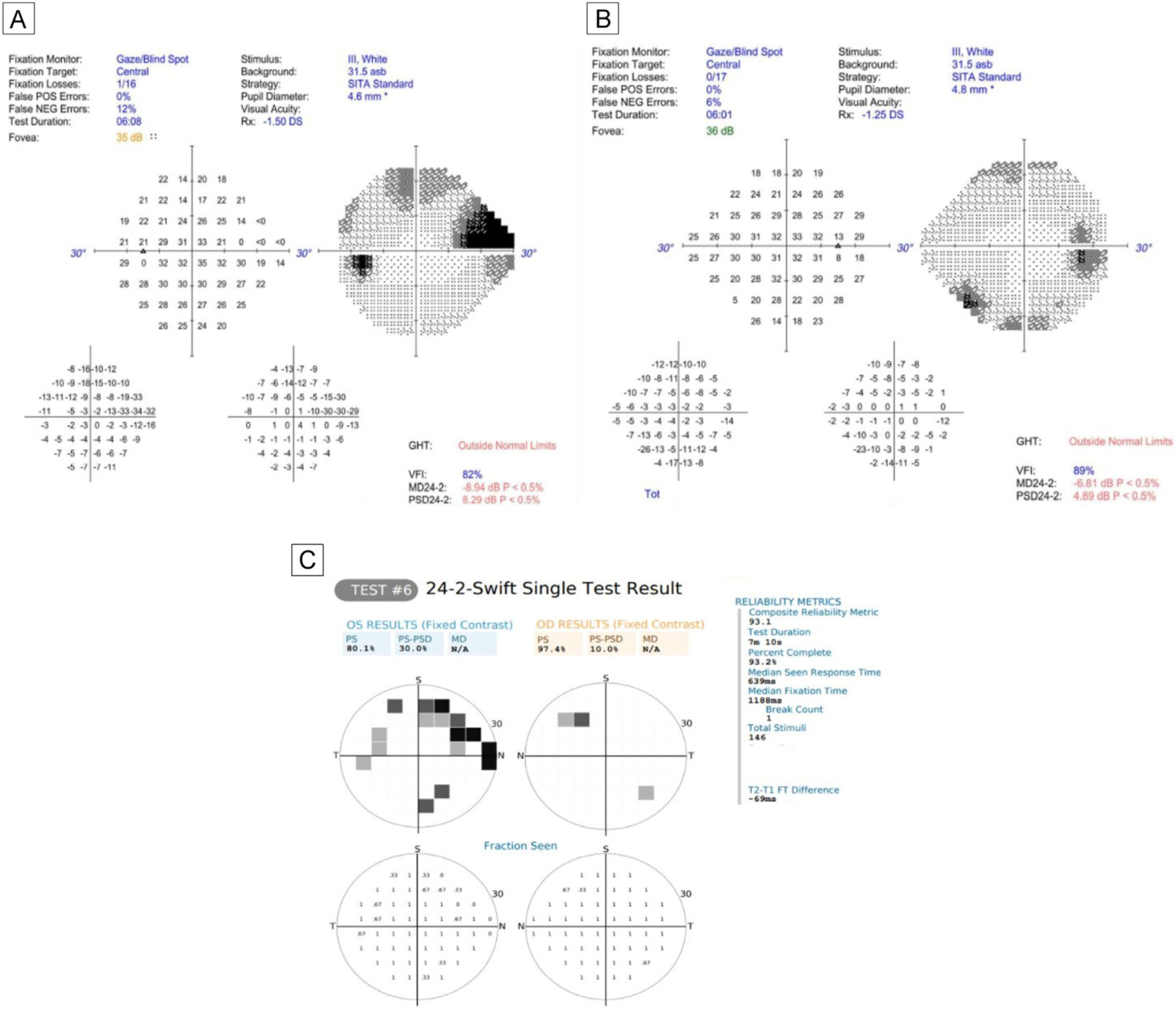
Example of Humphrey visual field (HVF) and VVP results. HVF 24-2 Standard results of a participant’s left eye and right eyes (A and B, resp.). The left eye demonstrates a superior arcuate visual field defect. The right eye demonstrates an early inferior arcuate visual field defect. C, VVP 24-2 Swift results of the same participant showing both eyes. The left eye demonstrates a superior arcuate defect similar to that seen in the HVF.

**FIG 3.**
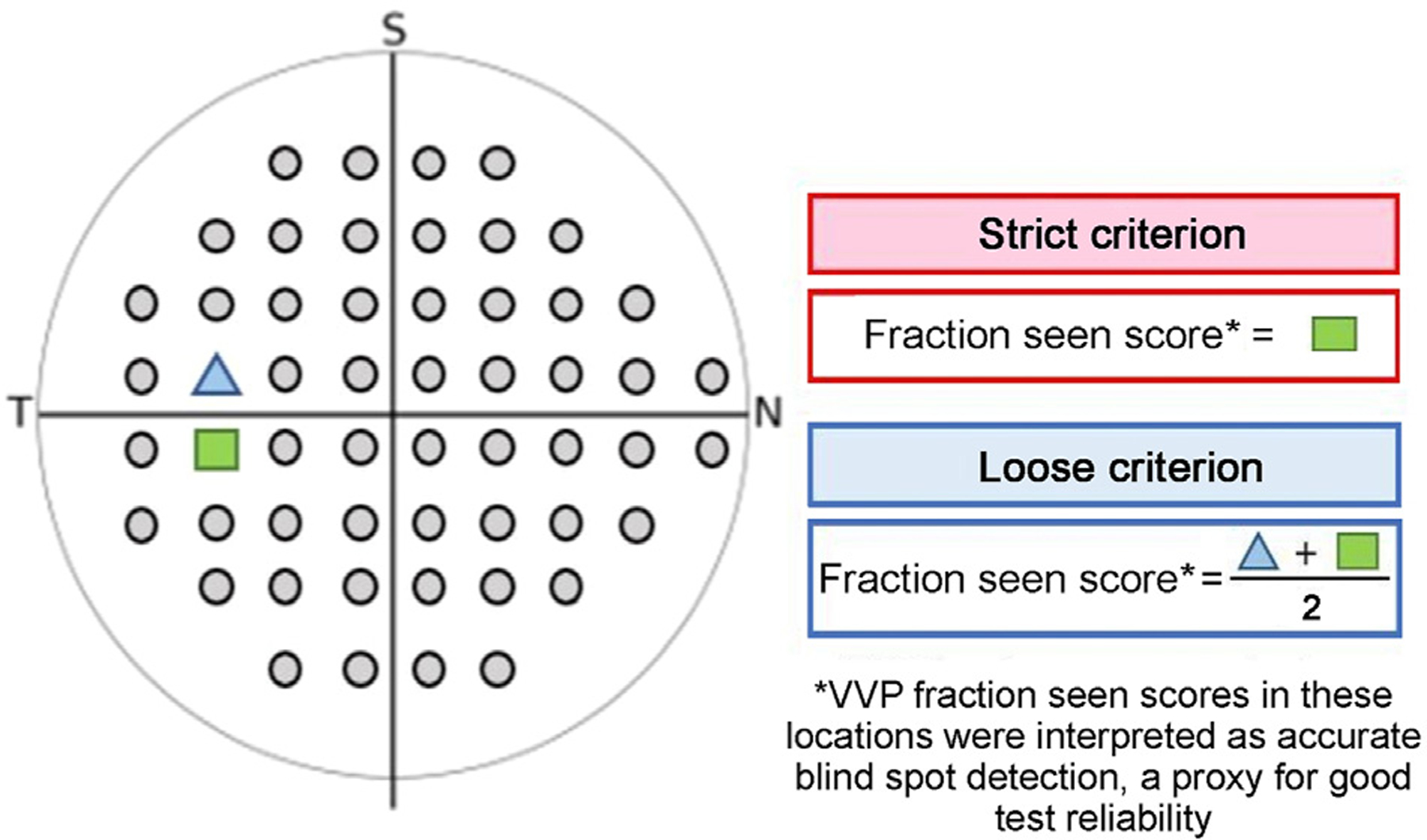
VVP reliability criteria. Schematic of the left eye demonstrating parameters used to assess VVP reliability using blind spot detection.

Screen mirroring with Scrpy software v1.25 displayed the VR device’s screen onto a laptop to monitor the patient’s progress. During the examination, VVP presented stimuli in 54 testing locations that corresponded to HVF 24-2’s strategy. The patient controls the position of the headset’s cursor using head movements (Figure 1). Before each stimulus is presented, a fixation task, in which the cursor must overlap with a fixation target, is completed to ensure proper fixation. Next, a peripheral flashing stimulus is presented. If the stimulus is seen, the patient moves their head to guide the cursor in the direction of the perceived stimulus and a stimulus is not presented again in the same location. If a stimulus is not seen, two additional stimuli are presented at the same testing location at different timepoints before the location is recorded as not seen. The final output includes the fraction seen score between 0–1 for each testing location, representing 0%−100%. Stimuli detected by the patient on their first attempt receive a fraction seen score of 1. The fraction seen across all 54 testing locations is averaged to calculate a mean fraction seen score and to map the patient’s VF (Figure 2). Because the VVP test does not have built-in reliability metrics, reliability was based on accurate blind spot detection according to two criteria (strict and loose). The strict reliability criterion was based on the score in one testing location associated with the patient’s anatomical blind spot. The loose reliability criterion was based on the score in two locations (Figure 3). Reliability was defined as a fraction seen score of ≤0.67 in the above locations, confirming that some stimuli in the anatomical blind spot were not seen. In a reliable test with perfect fixation, we would expect the fraction seen score to be 0 in the anatomical blind spot. Given the lack of manufacturer provided reliability metrics, we chose a cutoff of ≤0.67 to consider a test a reliable representation of a participant seeing 2 out of 3 or fewer stimuli presented in the blind spot.

After completing both tests, participants were surveyed. Participants provided information regarding prior experience with VR headsets, video games, HVF, VVP, or other VR-based perimetry. Participants ranked their satisfaction with VVP as “very satisfied,” “satisfied,” “neutral,” “dissatisfied,” or “very dissatisfied.” Lastly, participants were asked to express their personal preference between the two examinations with the question, “If you had to choose between the VVP and HVF tests, which exam would you prefer to take again?”

### Power Calculation and Statistical Analysis

Based on previous studies testing VVP in adults, a minimum sample size of 21 eyes was required to achieve 80% power (*β* = 0.2) and moderate correlation coefficient (*r* = 0.58) assuming a 5% significance level (*α* = 0.05).^4^ A Pearson correlation coefficient was calculated to compare the HVF mean sensitivity with the VVP mean fraction seen score. Statistical analysis was performed using R version 4.0.4 (R Foundation for Statistical Computing, Vienna, Austria).

## Results

Of the 24 eligible participants during the study enrollment period, all 24 were consented to participate, but only 23 were enrolled, with 1 child refusing to complete the VVP because of testing fatigue. A total of 37 eyes of 23 participants met inclusion criteria (9 eyes were excluded because of poor visual acuity or manifest strabismus). Mean age was 12.9 ± 3.1 years, and 48% of participants identified as female. Twelve participants were diagnosed with glaucoma, 7 with glaucoma suspect, 3 with ocular hypertension associated with steroid use, and 1 had a craniopharyngioma. Two participants were administered the HVF SITA Standard test, while the remaining 21 participants received the HVF SITA Fast. Mean logMAR visual acuity was 0.08 ± 0.17, and HVF mean deviation was −4.39 ± 5.9 dB. Participant demographics are provided in Table 1. Of our 23 participants, 15 (65%) had previously undergone HVF testing; none had prior VVP or other VR-based diagnostic testing experience, although 7 (30%) had exposure to VR through video games (Table 2).

All enrolled participants successfully completed the VVP tutorial and examination independently. VVP reliability was 54% and 65% by strict and loose criteria, respectively. Similarly, 65% of HVF tests were reliable. Only 5 patients had both unreliable HVF and VVP tests. Of the unreliable HVF examinations, 10 were due to fixation losses, 3 to false negatives, and 1 to both fixation losses and false positives. Mean response time for correctly seen stimuli on VVP was 0.71 ± 0.13 seconds and 0.91 ± 0.37 seconds for missed stimuli, defined as the fastest response time observed during the second or third attempt at testing a missed location. This value is not calculated if a participant misses all three presentations of a stimulus at a location. See Table 3.

**FIG 4.**
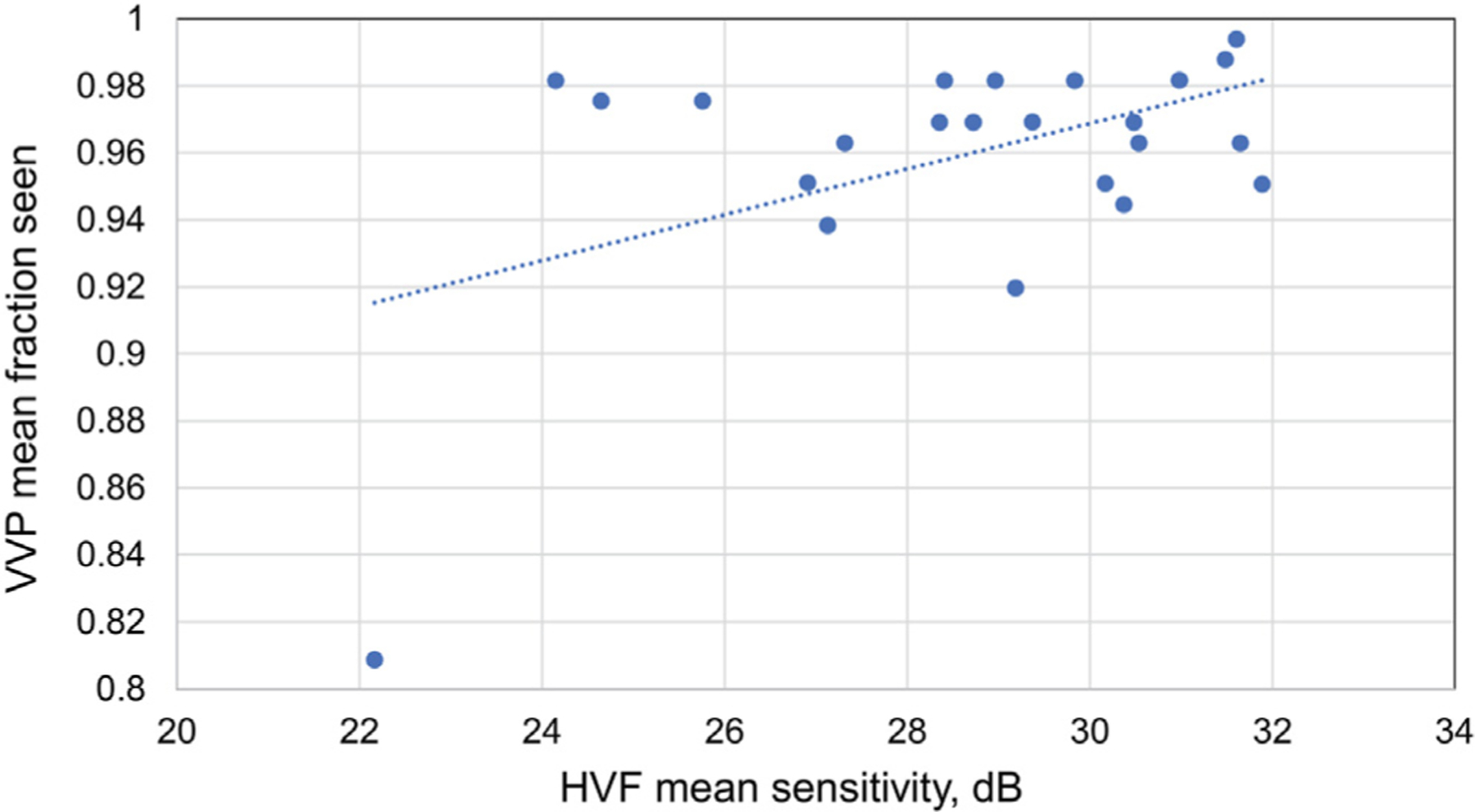
Correlation between HVF mean sensitivity and VVP mean fraction seen score.

Excluding unreliable HVF results, the correlation between HVF mean sensitivity and VVP fraction seen score was moderate (*R* = 0.48; *P* = 0.02; 95% CI, 0.09, 0.74). A significant relationship was not observed when excluding unreliable VVP results (*R* = 0.32; *P* = 0.12; 95% CI, −0.09 to 0.64), when including only reliable HVF and reliable VVP results (*R* = −0.25; *P* = 0.35; 95% CI, −0.67 to 0.28), or when including all VVP and HVF results (*R* = 0.05; *P* = 0.76; 95% CI, −0.27 to 0.37). See Table 4 and Figure 4.

All participants expressed a preference for VVP over HVF (Table 2). Approximately 70% were “very satisfied,” 26% “satisfied,” and 4% “neutral” regarding their VVP experience. No participants were dissatisfied with VVP (Table 2).

## Discussion

Overall, we found high feasibility and acceptance of VVP in children. All enrolled participants successfully completed VVP and expressed preference for it over the HVF test, highlighting its acceptability among children. This can possibly be attributed to greater familiarity and ease with the VR technology compared to traditional VF testing. Additionally, our study suggested some correlation between VVP and reliable HVF test results.

Despite being considered the gold standard for VF testing, several aspects of the HVF limit testing reliability in children. Despite the majority of participants having previous HVF experience, over 30% of them had unreliable HVF tests. By contrast, no participant had prior VVP experience, yet 65% produced reliable tests and all successfully completed the examination on their first attempt. Fixation losses were the most common reason for unreliable HVF tests, consistent with prior reports attributing it to the difficulty of suppressing one’s foveation reflex.^6,7^ Though the reliability rates as defined in our study for each test were found to be similar between the exams, the multi-fixation strategy employed by several VR perimeters may potentially make VR testing easier for children to understand. For example, Saccadic Vector Oculokinetic Perimetry, a computerized oculokinetic perimetry platform, has correlated with HVF results among children.^8^ VR represents a contemporary approach toward VF testing, and in children, other VR devices, such as the Olleyes VisuALL, have also demonstrated good results.^9^

VR-based visual field testing also addresses another shortcoming of HVF testing: patient positioning. In prior studies, adult patient anxiety during testing was significantly higher than pretest levels and correlated with test unreliability.^10^ A VR-based test allows the examinee to sit or stand in a position most comfortable to them and turn their heads rather than maintaining the same head position. Additionally, VR-based testing can decrease fidgeting and inattention, commonly seen in children, which contributes to high false positives during HVF testing.^6^ With VVP, false positives are not applicable, but engagement with VR could theoretically increase testing reliability in a patient population with highly variable measurements (Mahdavianim S, et al. IOVS 2008;49:ARVO eAbstract 1082).

VR-based testing has been associated with consistent patient satisfaction among adults, similar to our findings and those of the Olleyes VisuALL in children (Groth SL, et al. IOVS 2021;62:ARVO eAbstract 3391).^11^ For children, VR headsets offer some familiarity given the prevalence of these devices in the video game industry.^12^ Even among children who have no experience with VR, their knowledge of this technology can be leveraged in clinical practice. The unanimous preference for the VVP in our study cohort highlights its potential for increasing testing feasibility in this age group.

This pilot study demonstrated only a weak correlation between VVP mean fraction seen and mean sensitivity in reliable HVF tests, likely reflecting differences in the testing strategies. The HVF 24-2 SITA strategy tests each of the 54 locations more times than VVP 24-2 Swift and also at incremental intensities to calculate the patient’s threshold sensitivity. VVP 24-2 Swift, however, is a suprathreshold test that measures each location only once with a fixed contrast stimulus unless the stimulus is missed, such that in the absence of VF defects, most locations have a fraction seen value of 1. Thus, in this study population, with a high number of normal HVF tests, mean fraction seen showed limited variation, and it is not surprising that this metric, which would show greater variation in the setting of manifest VF defects, did not correlate well with HVF mean sensitivity. VVP strategies using mixed contrast stimuli have been developed but have not yet been tested in children (see, eg, www.perimetry.seevividly.com).^4^ Additionally, we only found a significant correlation between HVF and VVP when excluding unreliable HVF tests (not when excluding unreliable VVP tests or including all tests). This is likely a reflection of our VVP reliability criteria, which may not reflect true test reliability. This is an area for improvement and ideally something that will be integrated into the testing platform in future iteration.

Our study has several limitations. First, our small sample size limits generalizability and was not powered for subgroup analysis. Additionally, most participants presented with relatively normal VFs and due to differences in testing strategies, a direct comparison between the VVP and HVF may not be appropriate. For similar reasons, a pointwise comparison was also not performed. Second, although definitive reliability measures are in development, no reliability metrics currently exist for VVP. Thus, the blind spot method described here may not provide a comprehensive measure of reliability, and our decision to define reliable fixation as a fraction seen of up to 2/3 of blind spot presentations may be considered less stringent than a fixation loss rate criterion of <20% for HVF. Both testing platforms also demonstrated relatively high rates of unreliable tests, which also may have weakened the statistical weight of the correlation results. Additionally, though the participants were asked to express a preference between the VVP and HVF, they were not surveyed directly regarding their experience with the HVF, which may have biased their preferences. Similarly, all participants were tested with the HVF first, which may have biased subjects to report a stronger preference for VVP. As this technology continues to evolve, future studies can compare similar testing strategies in children with known visual field defects.

## Figures and Tables

**Table 1.** Demographic and ocular characteristics of study participants

Study parameter	Result
Number of participants approached	24
Number of participants enrolled	23
Number of eyes included	37
Age, years, mean ± SD	12.9 ± 3.1
Sex (% female)	47.8
Self-identified race/ethnicity, no. (%)	
White	3 (13.0)
Asian	9 (39.1)
Black	2 (8.7)
Latinx	4 (17.4)
Other	3 (13.0)
Declined to answer	2 (8.7)
Diagnosis, no. (%)	
Glaucoma	12 (52.1)
Glaucoma suspect	7 (30.4)
Steroid-induced ocular hypertension	3 (13.0)
Craniopharyngioma	1 (4.3)
LogMAR visual acuity (mean ± SD)	0.08 ± 0.17

*LogMAR*, logarithm of the minimum angle of resolution; *SD*, standard deviation.

**Table 2.** Post-testing survey results

Survey question	Percentage (n = 23)
Previous experience with HVF	65.2
Previous experience with VVP or other VR perimetry	0
Previous experience with VR games	30.4
VVP Satisfaction	
Very satisfied	69.5
Satisfied	26.0
Neutral	4.3
Dissatisfied	0
Very dissatisfied	0
Patient exam preference	
HVF	0
VVP	100

*HVF*, Humphrey visual field; *VR*, virtual reality; *VVP*, Vivid Vision Perimetry.

**Table 3.** HVF and VVP test results and reliability metrics

Study parameter	Result
HVF 24-2 SITA Fast and Standard	
Number of HVF 24-2 SITA Standard tests, no. (%)	2 (8.7)
Mean test duration per eye, minutes, mean ± SD	3.90 ± 1.00
Mean deviation, dB, mean ± SD	−4.39 ± 5.9
Mean sensitivity, dB, mean ± SD	27.9 ± 5.6
Total unreliable tests, no. (%)	14 (37.8)
>15% fixation losses	11 (29.7)
>30% false negatives	3 (8.1)
>30% false positives	1 (2.7)
VVP 24-2 Swift	
Mean test duration per eye, minutes, mean ± SD	3.91 ± 1.34
Mean fraction seen, mean ± SD	0.96 ± 0.04
Mean seen response time, seconds, mean ± SD	0.71 ± 0.13
Mean incorrect response time, seconds, mean ± SD	0.91 ± 0.37
Total unreliable tests-strict criteria, no. (%)	17 (45.9)
Total unreliable tests-loose criteria, no. (%)	13 (35.1)

*HVF*, Humphrey visual field; *SD*, standard deviation; *SITA*, Swedish interactive testing algorithm; *VVP*, Vivid Vision Perimetry.

**Table 4.** Correlation between HVF and VVP as a function of test reliability

Correlation test	*R* ^a^	*P* value	95% CI
Reliable HVF vs VVP	0.48	0.02	0.09 to 0.74
Reliable VVP vs HVF	0.32	0.12	−0.09 to 0.64
Reliable HVF vs reliable VVP	−0.25	0.35	−0.67 to 0.28
All HVF vs all VVP	0.05	0.76	−0.27 to 0.37

*HVF*, Humphrey visual field; *VVP*, Vivid Vision Perimetry.

a Pearson correlation.
